# Case Report: Pulmonary sclerosing pneumocytoma mimicking as a neuroendocrine tumor on ^18^F-FDG and ^68^Ga-DOTATATE PET/CT: a case presentation

**DOI:** 10.3389/fonc.2025.1511595

**Published:** 2025-02-21

**Authors:** Ronghua Yu, Wei Zhao, Yonglin Yu, Xianwen Hu

**Affiliations:** ^1^ Affiliated Hospital of Zunyi Medical University, Department of Nuclear Medicine, Zunyi, China; ^2^ Department of Pathology, Affiliated Hospital of Zunyi Medical University, Zunyi, China

**Keywords:** pulmonary sclerosing pneumocytoma, lung carcinoid, ^18^F-FDG, PET/CT, ^68^Ga-DOTATATE

## Abstract

Pulmonary sclerosing pneumocytoma (PSP) is a relatively rare benign lung tumor, and it is difficult to obtain an accurate diagnosis before surgery. Herein, we present a case of 34-year-old woman who came to our hospital for medical help due to cough and sputum for one month. She underwent a chest computed tomography (CT) scan which revealed a circular soft tissue density shadow in the upper lobe of the left lung. A needle biopsy was subsequently performed which revealed a probable lung carcinoid. To further evaluate the nature of the mass and determine a treatment plan, the patient subsequently underwent dual nuclide tracer including fluorine-18 labeled fluorodeoxyglucose (^18^F-FDG) and gallium-68 labeled 1, 4, 7, 10-tetraazacyclododecane-1, 4, 7,10-tetraaceticacid -D-Phel-Tyr3-Thr8-OC (^68^Ga-DOTATATE) PET/CT imaging. The results showed that the lession presented increased both ^18^F-FDG and ^68^Ga-DOTATATE uptake, suggesting a neuroendocrine tumor. However, postoperative pathology confirmed that the lesion was PSP. Our case study suggests that PSP may presents varying degrees of increased ^18^F-FDG and ^68^Ga-DOTATATE uptake on positron emission tomography (PET)/CT imaging, which should be considered as one of the differential diagnoses for lung carcinoids.

## Introduction

Pulmonary sclerosing pneumocytoma (PSP) was previously thought to originate from endothelial and vascular tissue and was called sclerosing hemangioma. However, it was later shown to originate in type II alveolar epithelial cells, so in 2015 the World Health Organization renamed it PSP (1). PSP is most common in middle-aged women in Asia, where the incidence of women is five times that of men (2). PSP usually has no obvious clinical symptoms and is usually found by chance during routine imaging examinations. Some patients could complain of cough and chest and back pain, which may be caused by tumor compression of surrounding tissues (3). Due to the relative rarity of PSP and the non-specific clinical symptoms, it is difficult to obtain an accurate diagnosis before surgery, especially when the imaging findings are atypical. Herein, we present the diagnosis and treatment of a patient with PSP, focusing on the presentation of it on dual nuclide tracers including fluorine-18 labeled fluorodeoxyglucose (^18^F-FDG) and gallium-68 labeled 1, 4, 7, 10-tetraazacyclododecane-1, 4, 7,10-tetraaceticacid -D-Phel-Tyr3-Thr8-OC (^68^Ga-DOTATATE) positron emission tomography (PET)/computed tomography (CT) to increase the understanding of this relatively rare benign lung tumor.

## Case presentation

**Figure 1:**
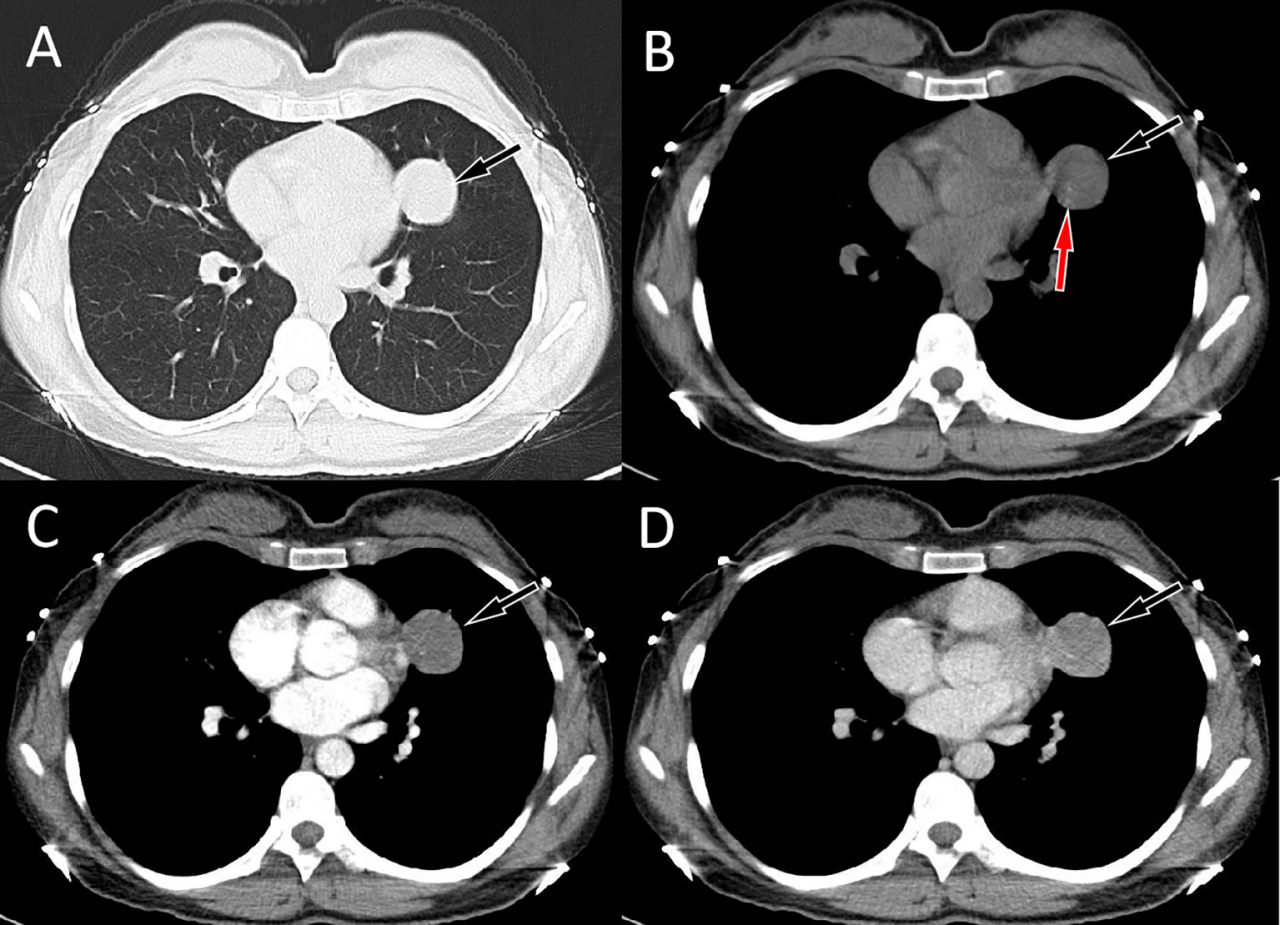
Chest computed tomography (CT) pulmonary window **(A)** and mediastinal window **(B)** revealed a soft tissue density mass (black arrows) about 3.4 cm×3.2 cm in size with punctate calcification (red arrow) can be seen on the upper lobe of the left lung; In the arterial phase **(C)** of contrast-enhanced CT, the lesion showed mild uniform enhancement (arrow); and in the vein phase **(D)**, the lesion showed continuous uniform enhancement (arrow), and the degree of enhancement increased compared with the arterial phase.

**Figure 2:**
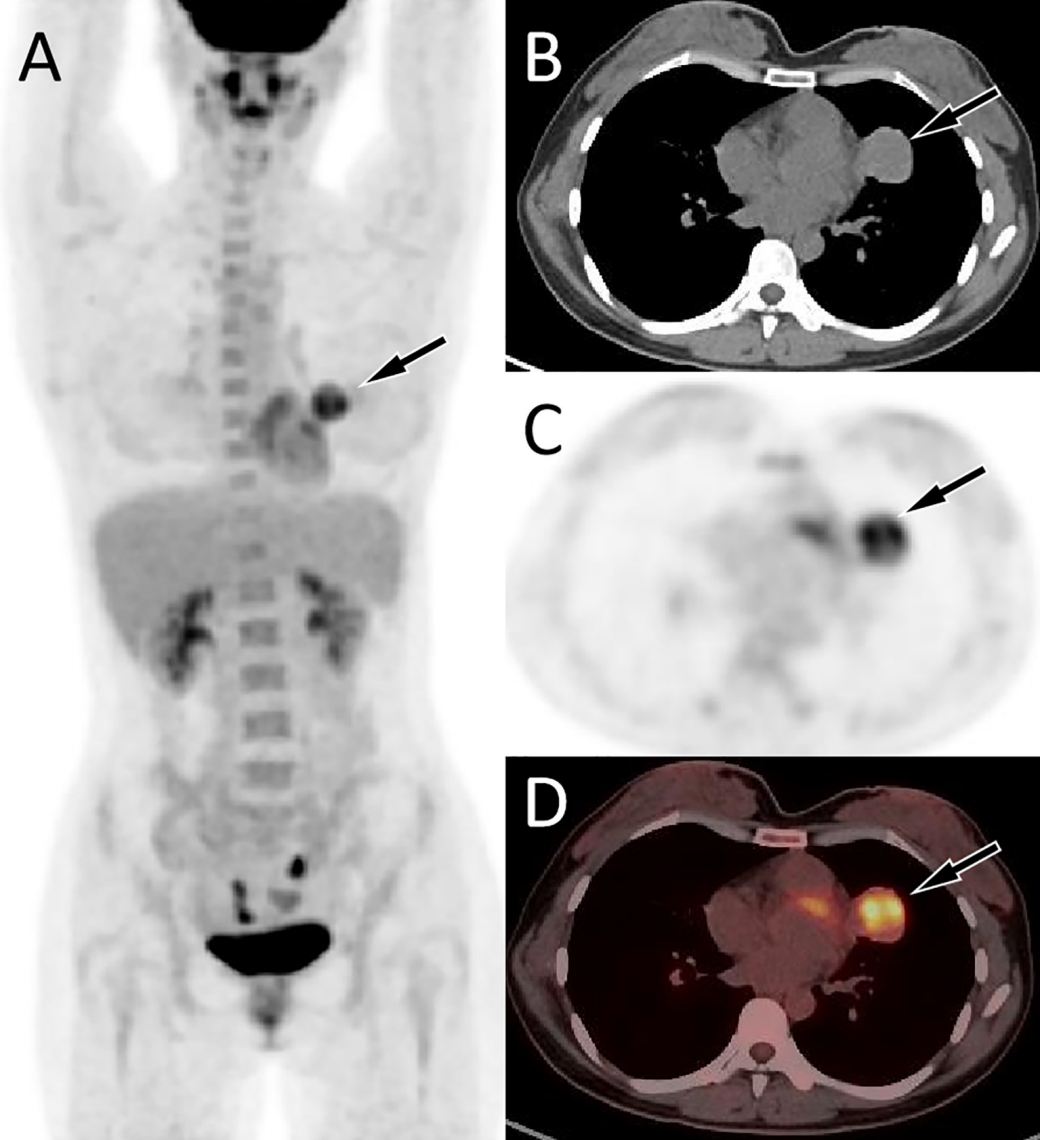
Fluorine-18 fluorodeoxyglucose (^18^F-FDG) positron emission tomography (PET)/CT imaging of the patient; The maximum intensity projection [MIP, **(A)**] showed an increased ^18^F-FDG uptake in the left upper lung (arrow). Axial CT **(B)** showed a soft tissue mass in the upper lobe of the left lung (arrow). The corresponding lesion presented increased ^18^F-FDG uptake on axial PET [**(C)**, arrow] and PET/CT fusion [**(D)** arrow], with a maximum standardized uptake value (SUVmax) of 5.6.

**Figure 3:**
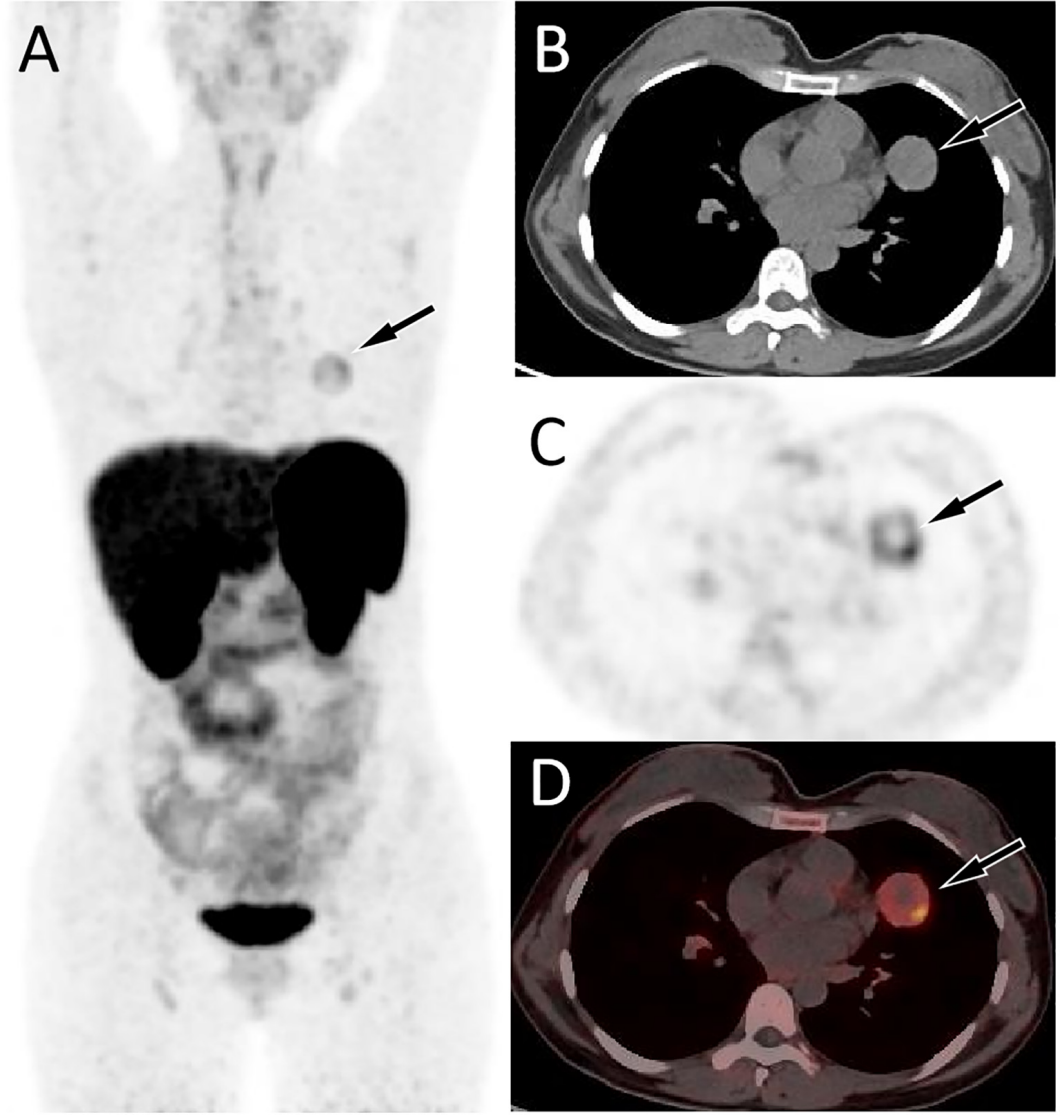
^68^Ga-DOTATATE PET/CT imaging of the patient; The MIP **(A)** showed an increased 68Ga-DOTATATE uptake in the left upper lung (arrow). Axial CT **(B)**, PET **(C)** and PET/CT fusion **(D)** showed this inhomogeneously increased focal uptake in the upper lobe of the left lung (arrow), with lower uptake areas in the center zome of the lesion due to necrotic changes, in the same location as the lesion shown on ^18^F-FDG PET/CT, with a SUVmax of 4.4.

**Figure 4:**
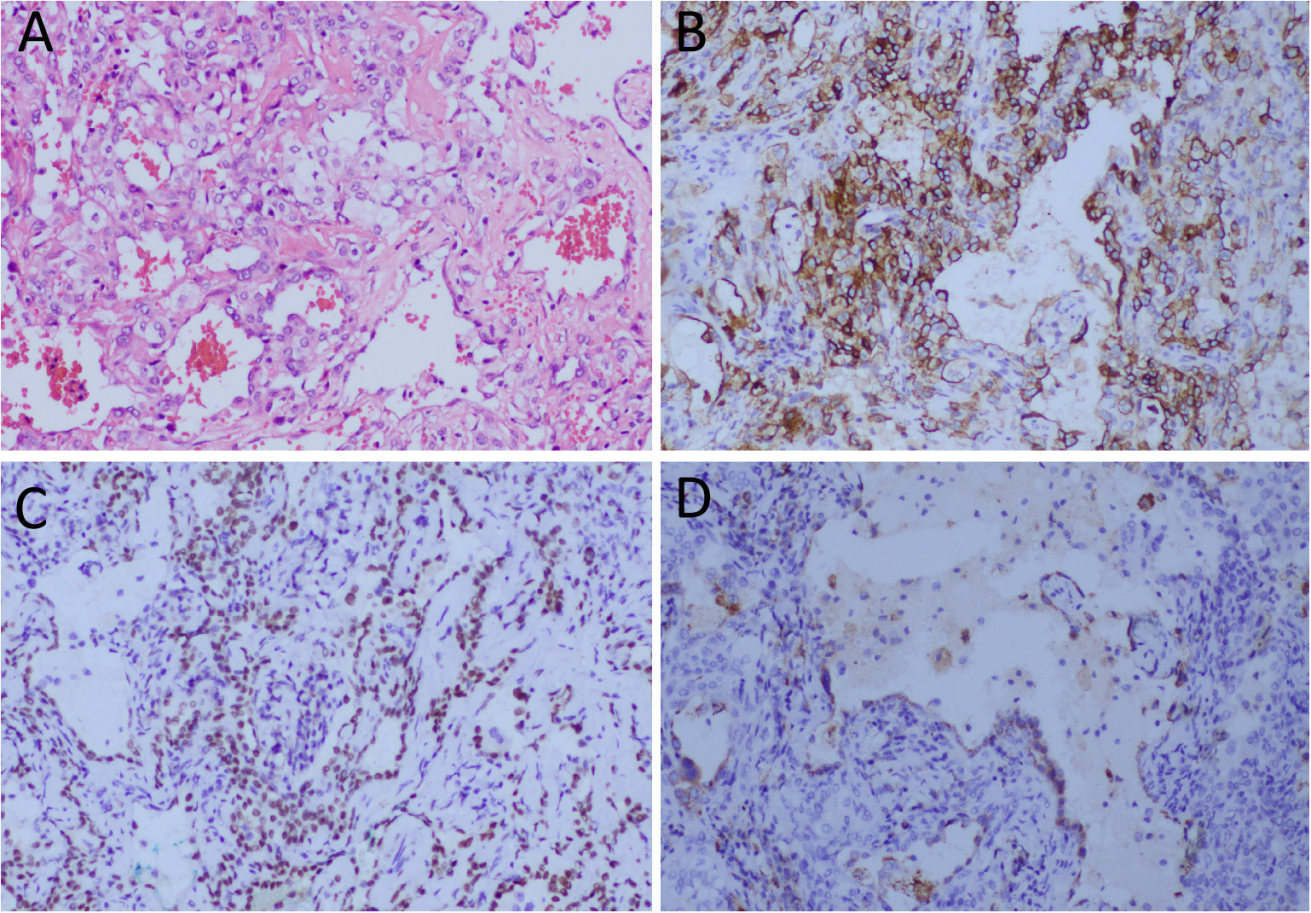
**(A)** Hematoxylin-eosin staining showing the cubed epithelial cells on the surface of the papillary structure and the rounded stromal cells between the solid area and the papillary structure are seen within in the tumor tissue. Immunohistochemical results showed that the tumor cells positively expressed EMA **(B)**, TTF1 **(C)** and Napsin-A **(D)**. Notes: SMA, epithelial membrane antigen; TTF1, thyroid transcription factor 1; All images are 100 times magnification.

A 34-year-old woman came to our hospital for medical help due to cough and sputum for one month. Now the patient has no other discomfort except cough and sputum. Physical examination revealed that no abnormal breathing sounds were heard in the patient’s lungs, and no positive signs such as tenderness, pleural friction sound and subcutaneous superficial lymph node enlargement were found. She and her family have denied any history of cancer or genetic disease, tuberculosis, hepatitis and other infectious or serious illnesses. Her blood routine, serum tumor markers and other laboratory results were negative. The patient underwent a chest CT scan, which showed a circular soft tissue density shadow in the upper lobe of the left lung, with spotted high-density calcifications visible inside. The lesion showed mild enhancement on contrast-enhanced CT scan (as shown in **Figure 1**). The patient subsequently underwent a CT-guided needle biopsy, which indicated the possibility of a neuroendocrine tumor, pulmonary carcinoid. To further evaluate the nature of the mass and determine a treatment plan, the patient subsequently underwent dual nuclide tracer including ^18^F-FDG (**Figure 2**) and ^68^Ga-DOTATATE PET/CT (**Figure 3**) imaging (The interval between the two tracers was 24 hours). The results revealed that the lession presented inhomogeneously increased both ^18^F-FDG and ^68^Ga-DOTATATE uptake. The peripheral area of the lesion showed higher uptake, while the central area of the lesion showed lower uptake, possibly due to necrosis. In addition, no hot spot lesions were observed in the rest of the body. Based on these imaging findings, the patient was initially suspected to have a carcinoid. Because the lesion was isolated and localized, the patient underwent surgical resection of the mass after a series of evaluations. Hematoxylin eosin staining (as shown in **Figure 4**) showed matrixes composed of circular cells within the tumor, with epithelial structures composed of cubic cells covering the surface, and some gaps filled with red blood cells. Immunohistochemical results showed that the tumor cells positively expressed epithelial membrane antigen (EMA), thyroid transcription factor 1 (TTF1), NapsinA (epithelial cells) and cytokeratin 7 (epithelial cells), while negatively expressed chromogranin A (CgA) and synaptophysin (Syn). Based on the morphology of the tumor under the microscope and immunohistochemical findings, the patient was ultimately diagnosed with PSP. Due to the benign tumor nature of PSP, the patient was discharged after 3 days of anti-inflammatory treatment following surgery. The patient has been followed for 9 months and she has not complained of any discomfort.

## Discussion

PSP is a relatively rare benign tumor, accounting for only 2% to 3% of lung tumors, and is most common in women between the ages of 40 and 60 (4). Most PSP patients have no obvious clinical manifestations, and a few can present cough, sputum, shortness of breath, hemoptysis, chest pain, chest tightness and other atypical clinical manifestations (5). The patient we present is female, but younger than the preferred age for PSP. Due to cough and phlegm, she subsequently underwent a chest CT examination which revealed a lung nodule.

Previous imaging studies on PSP have focused on CT findings. Most of the CT findings of PSP show the imaging features of benign lung tumors with isolated masses or nodules with smooth edges and clear boundaries, which may be accompanied by calcification and cystic degeneration (6). Especially, the typical CT signs of PSP include vascular patching sign, air crescent sign, pulmonary artery main sign, tail sign, halo sign and so on (7, 8). On contrast enhanced CT scan, different degrees of enhancement can occur according to the different size of the lesion and the proportion of tissue components, and progressive and delayed continuous enhancement are the relative characteristics (9). The CT findings of the patient we reported showed a circular soft tissue density nodule with a small amount of calcification. The contrast enhanced scan showed moderate enhancement, which was characteristic of a benign lung tumor, but could not exclude the possibility of lung carcinoid cancer. Due to the benign tumor nature of PSP, there have been few studies on PET/CT imaging of it. ^18^F-FDG is one of the most commonly used tracers for PET-C, and its level of energy uptake often reflects the malignancy of the tumor. Studies have shown that PSP usually presents with a slightly to moderately increased ^18^F-FDG uptake, the intensity of uptake is positively correlated with tumor size, and symptomatic patients show a higher average uptake of ^18^F-FDG than asymptomatic patients (10, 11). The patient in our current report showed significantly increased ^18^F-FDG uptake on PET/CT, which may be related to the relatively large tumor size (greater than 3.0cm). Moreover, the patient underwent ^68^Ga-DOTATATE PET/CT imaging due to the high probability of the needle biopsy indicating lung carcinoid cancer. Lung carcinoids are neuroendocrine tumors (NET), most of which express somatostatin receptors (SSTR), especially type 2. ^68^Ga-DOTATATE has a relatively high affinity for SSTR and can be used as a tracer in PET/CT imaging for targeted diagnosis of SSTR. It can not only determine the location of the primary tumor, but also detect some small metastases that are difficult to find, which is of great significance for improving the accuracy of NET diagnosis and determining the tumor stage, and thus improving the prognosis (12, 13). However, interestingly, although the patient we reported was ultimately diagnosed with PSP, non NETs, it also showed increased ^68^Ga-DOTATATE uptake on somatostatin receptor PET/CT imaging. Due to the rarity of similar studies in the past, the reasons for the increased ^68^Ga-DOTATATE uptake in PSP need further investigation.

According to the imaging findings and clinical features of PSP, it should be distinguished from pulmonary carcinoid, peripheral pulmonary adenocarcinoma, tuberculous spheres. Lung carcinoids, especially peripheral carcinomas, often appear as solitary round or quasi-round soft tissue masses on CT. About 30% of the tumors may have spotty or lamellar calcifications, showing moderate to significant enhancement on contrast-enhanced CT (14). Moreover, lung carcinoids may also show varying degrees of increased uptake of ^18^F-FDG or ^68^Ga-DOTATATE on PET imaging (15, 16), making it difficult to distinguish them from PSP. A previous study revealed that the “iceberg sign”, that is, the small tumor is manifested as a bronchial lumen lesion, or the small bronchial lumen nodule is fused with a large extracellular lung lesion, is a characteristic manifestation of lung carcinoid (14), and can be distinguished from other diseases, but the occurrence rate of this sign is rare. Peripheral lung adenocarcinoma often shows burrs, lobulation, pleural depression, and less calcification, which often shows obvious uneven enhancement on contrast-enhanced scan, and usually significantly increased ^18^F-FDG uptake on PET imaging (17, 18). However, PSP usually presents a round-like soft tissue mass, which usually shows uniform enhancement on contrast-enhanced scan, and there was a mildly to moderately increased ^18^F-FDG uptake on PET, so identification of the two was relatively easy. Tuberculomas can have fever, night sweat and other toxic symptoms, and CT mostly also shows circular nodules with calcification and necrosis, while it is often accompanied by satellite foci around, and the enhancement degree of tuberculomas is weaker than that of PSP on contrast-enhanced CT scan (19). Other differential diagnoses include hamartoma, inflammatory pseudotumor, adenoma and fungal infection, which can be distinguished according to the clinical manifestations and imaging characteristics of patients.

The diagnosis of PSP is confirmed by histopathological examination. Under the microscope, the tumor tissue structure of PSP is relatively complex, including four regions consisting of nipple area, solid area, hardened area, and hemorrhagic area, as well as two basic types of tumor cells: surface cuboidal epithelial cells and circular stromal cells (20). Immunohistochemical results showed that TTF-1 and EMA were positive in epithelial and stromal cells of almost all tumor tissues (2). Moreover, CK7 and Napsin-A, CD56, Syn were also expressed positively in some tumor tissues (21). The pathological features of this patient reported by us were consistent with the above literature, and both epithelial cells and stromal cells of the tumor had positive expressions of EMA and TTF1, and CK7 and Napsin-A were also positively expressed in epithelial cells.

The treatment of PSP is mainly thoracoscopic resection, as the nature of the benign tumor, further mediastinal lymph node dissection is not necessary (5). The prognosis of PSP after surgical resection is generally good, although mediastinal lymph node metastasis, pleural metastasis and even distant metastasis have been reported in the literature, but it does not affect the survival of patients (22–26). There was no evidence of tumor recurrence after surgical resection of the tumor in the patient we reported at present. The most recent telephone follow-up was 9 months after discharge, and the patient did not report any discomfort.

## Conclusion

PSP is a relatively rare benign lung tumor, and it is difficult to obtain an accurate diagnosis before surgery. Our case study suggests that PSP presents varying degrees of increased ^18^F-FDG and ^68^Ga-DOTATATE uptake on PET/CT imaging, which should be considered as one of the differential diagnoses for lung carcinoids.

## Data Availability

The original contributions presented in the study are included in the article/supplementary material. Further inquiries can be directed to the corresponding authors.
